# The glucosyltransferase activity of *C. difficile* Toxin B is required for disease pathogenesis

**DOI:** 10.1371/journal.ppat.1008852

**Published:** 2020-09-22

**Authors:** Terry W. Bilverstone, Megan Garland, Rory J. Cave, Michelle L. Kelly, Martina Tholen, Donna M. Bouley, Philip Kaye, Nigel P. Minton, Matthew Bogyo, Sarah A. Kuehne, Roman A. Melnyk

**Affiliations:** 1 Clostridia Research Group, BBSRC/EPSRC Synthetic Biology Research Centre, School of Life Sciences, Centre for Biomolecular Sciences, The University of Nottingham, Nottingham, United kingdom; 2 Cancer Biology Program, Stanford School of Medicine, Stanford, CA, United States of America; 3 Department of Pathology, Stanford School of Medicine, Stanford, CA, United States of America; 4 Department of Comparative Medicine, Stanford School of Medicine, Stanford, CA, United States of America; 5 Department of Histopathology, Nottingham University Hospitals and University of Nottingham NDDC NIHR BRC, Nottingham, United Kingdom; 6 NIHR Nottingham Biomedical Research Centre, Nottingham University Hospitals NHS Trust and the University of Nottingham, Nottingham, United Kingdom; 7 Department of Microbiology and Immunology, Stanford School of Medicine, Stanford, CA, United States of America; 8 Oral Microbiology Group, School of Dentistry and Institute of Microbiology and Infection, College of Medical and Dental Sciences, The University of Birmingham, Birmingham, United Kingdom; 9 Department of Biochemistry, University of Toronto, and Program in Molecular Medicine, The Hospital for Sick Children, Toronto, ON, Canada; University of Pittsburgh School of Medicine, UNITED STATES

## Abstract

Enzymatic inactivation of Rho-family GTPases by the glucosyltransferase domain of *Clostridioides difficile* Toxin B (TcdB) gives rise to various pathogenic effects in cells that are classically thought to be responsible for the disease symptoms associated with *C*. *difficile* infection (CDI). Recent *in vitro* studies have shown that TcdB can, under certain circumstances, induce cellular toxicities that are independent of glucosyltransferase (GT) activity, calling into question the precise role of GT activity. Here, to establish the importance of GT activity in CDI disease pathogenesis, we generated the first described mutant strain of *C*. *difficile* producing glucosyltransferase-defective (GT-defective) toxin. Using allelic exchange (AE) technology, we first deleted *tcdA* in *C*. *difficile* 630Δ*erm* and subsequently introduced a deactivating D270N substitution in the GT domain of TcdB. To examine the role of GT activity *in vivo*, we tested each strain in two different animal models of CDI pathogenesis. In the non-lethal murine model of infection, the GT-defective mutant induced minimal pathology in host tissues as compared to the profound caecal inflammation seen in the wild-type and 630Δ*ermΔtcdA* (*ΔtcdA*) strains. In the more sensitive hamster model of CDI, whereas hamsters in the wild-type or *ΔtcdA* groups succumbed to fulminant infection within 4 days, all hamsters infected with the GT-defective mutant survived the 10-day infection period without primary symptoms of CDI or evidence of caecal inflammation. These data demonstrate that GT activity is indispensable for disease pathogenesis and reaffirm its central role in disease and its importance as a therapeutic target for small-molecule inhibition.

## Author summary

Novel non-antibiotic therapies are required for the treatment of *Clostridioides difficile* infection (CDI). An emerging class of promising therapeutics for CDI are antivirulence agents that block the actions of *C*. *difficile* Toxin B (TcdB), the primary determinant of virulence. In order to develop such treatments, molecular targets and mechanisms must be identified and validated. Historically the glucosyltransferase domain (GTD) represented an ideal target owing to its perceived importance for disease pathogenesis. However, studies capitalizing on recent advances in recombinant TcdB production have unveiled GTD-independent mechanisms of toxicity when applied at high concentrations *in vitro*, thus questioning the role of the GTD. Here we generate the first-reported mutant strain of *C*. *difficile* expressing glucosyltransferase-defective TcdB. Application thereof demonstrates that the GTD is essential for disease in mice and hamsters, thus reoffering the GTD as an ideal candidate for small-molecule inhibitor (SMI) development.

## Introduction

*Clostridioides difficile* (previously known as *Clostridium difficile* [1]) is the leading cause of nosocomial diarrhea in the developed world [2]. The main virulence factors responsible for the onset of symptoms during *C*. *difficile* infection (CDI), including diarrhea and pseudomembranous colitis, are the monoglucosyltransferases Toxin A (TcdA; 308kDa) and Toxin B (TcdB; 270 kDa) [3]. The application of *tcdA* and *tcdB-*null mutant strains to *in vivo* models, coupled with the frequent isolation of clinical isolates producing only TcdB, has consolidated the notion that–though TcdA can contribute to disease severity–TcdB represents the primary virulence determinant for CDI [4–6]. TcdB is therefore the primary target for the development of novel toxin-targeted therapeutics, including monoclonal antibodies and small-molecule inhibitors (SMIs).

TcdB is a single polypeptide cytotoxin composed of four distinct domains: a receptor-binding domain (RBD), a translocation domain (TD), an autoprocessing domain (APD), and a glucosyltransferase domain (GTD). TcdB is endocytosed into endosomes following receptor binding to one of three defined receptors: chondroitin sulfate proteoglycan 4 (CSPG4), Nectin 3 and frizzled protein (FZD1, 2 and 7) at least in part due to interaction with the RBD [7–9]. Thereafter, vesicular acidification leads to a conformational change and protein unfurling, ascribed to the hydrophobic region of the TD, permitting membrane insertion and the formation of an endosomal pore, through which the GTD and APD translocate into the cytosol [10]. Cytosolic inositol hexakisphosphate (InsP6) acts as an allosteric activator to initiate the cysteine-protease activity of the APD, which cleaves the GTD, leading to its release into the cytosol [11]. The GTD then glucosylates Rho-family GTPases leading to the classical phenotype of rapid actin depolymerization, loss of structural integrity, cell rounding and apoptosis of the colonic epithelium [12].

Over the past decade, significant efforts have been made to characterize the contribution of the GTD to disease pathogenesis through the generation of recombinant TcdB defective in glucosyltransferase activity (GT-defective). These studies have unveiled novel GT-independent mechanisms of toxicity for TcdB. Rapid necrotic cell death was observed independent of the GTD when GT-defective TcdB was applied *in vitro* at ≥1nM [13]. In the same study, an active GTD domain was required to induce the classical cytopathic effect at concentrations of <10pM. The necrotic mechanism was later characterized to be dependent on the host NADPH oxidase complex [14]. Thereafter, a second GT-independent mechanism of cell death was discovered to occur through a pyknotic mechanism [15]. In a similar fashion to the aforementioned necrotic mechanism, the pyknotic pathway was restricted to high concentrations of TcdB. Finally, two studies investigating TcdB-induced inflammasome activation, using wild-type TcdB and GT-defective TcdB, yielded opposing conclusions on the dependence of GT activity on inflammasome activation [16, 17]. Such discoveries question the contribution of the GTD to the pathogenesis of CDI and divert focus for SMI development away from the GTD. Ultimately, *in vivo* substantiation of the *in vitro* discoveries is needed to clarify the importance of GT activity in a natural disease setting. Previous attempts have been made to correlate the relevance of the GT-independent mechanisms of TcdB to infection. Therein, GT-defective TcdB was injected into mice [18], and in parallel, mice were infected with a surrogate *Bacillus megaterium* host strain, engineered to secrete GT-defective TcdB [19]. In neither instance was a GT-independent effect of virulence observed. These experiments question the relevance of the GT-independent mechanisms of TcdB-induced toxicity to infection, but hitherto, no study has been conducted with a strain of *C*. *difficile* engineered to produce full-length GT-defective toxin.

This study sought to clarify the role of the GTD to disease pathogenesis *in vivo* through the generation of a mutant strain of *C*. *difficile* devoid of GT-activity and its application to murine and hamster models of infection.

## Results and discussion

### Mutant strain authentication and *in vitro* characterization

**Fig 1:**
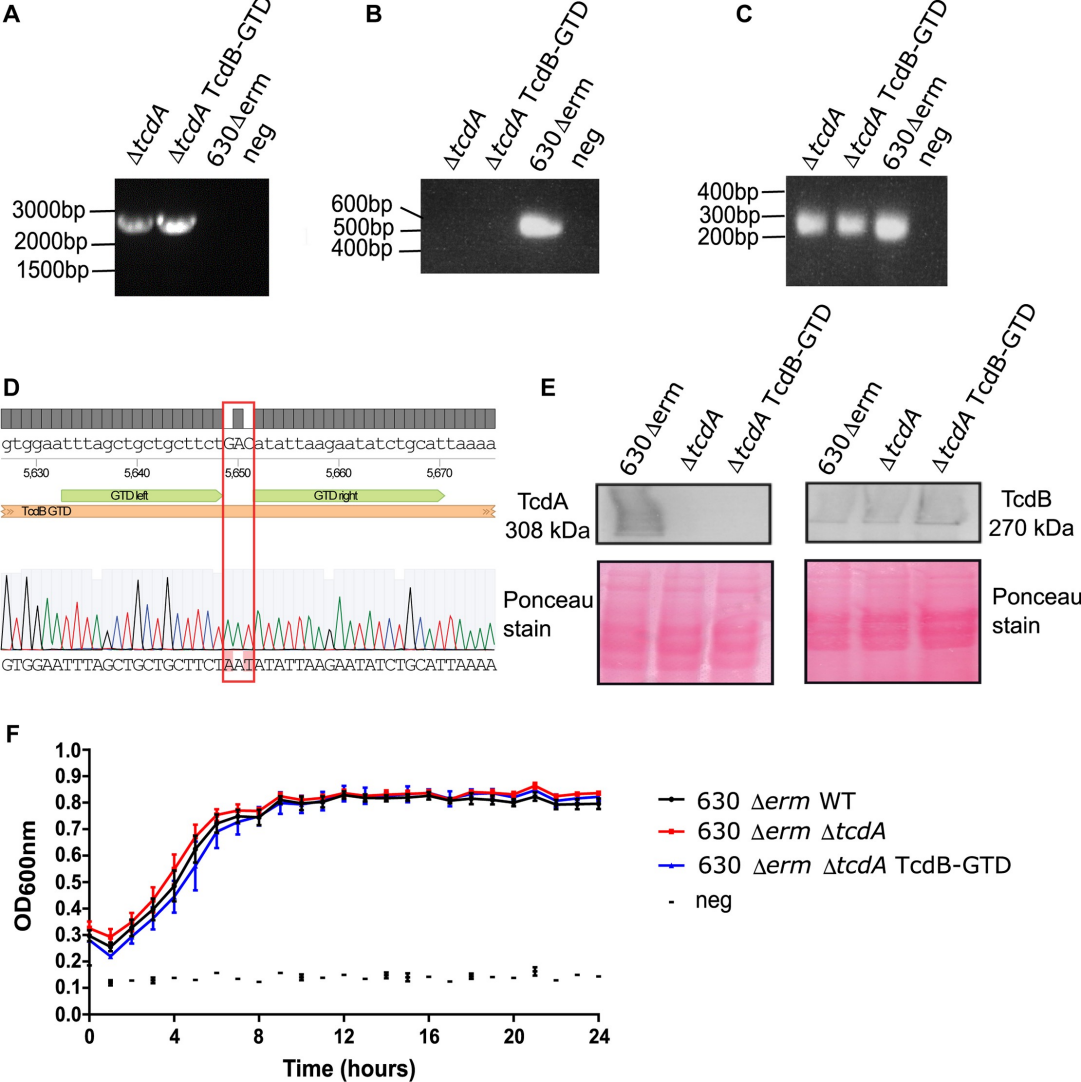
Development and authentication of a mutant strain producing GTD-defective TcdB. **a)** Gel image following PCR of the *tcdA* gene using the flanking primers *tcdA* diag F/*tcdA* diag R. The presence of a circa 2.7kb band indicates successful gene deletion. **b)** Gel image following PCR of the *tcdA* gene using the internal primers *tcdA* int F/*tcdA* int R. The absence of a 500bp product indicates successful gene deletion. **c)** Gel image following PCR of the GTD of *tcdB* using GTD diag F/R primers. Strain labels: *C*. *difficile* 630Δ*erm* (WT); *C*. *difficile* 630Δ*erm*Δ*tcdA* (*tcdA*); *C*. *difficile* 630Δ*erm*Δ*tcdA* GTD::D270N (GTD). **d)** Sequence alignment of the GTD region of *tcdB* for *C*. *difficile* 630Δ*erm*Δ*tcdA* GTD::D270N with the wild-type reference sequence of 630Δ*erm*. The desired gac-aat substitution is highlighted in a red box. **e)** Western blot detection for TcdA and TcdB for the wild-type, Δ*tcdA* and GTD-D270N strains including Poncaeu stains to validate even protein loading. **f)** 24h automated growth curve of the wild-type, Δ*tcdA* and GTD-D270N strains.

In an attempt to reconcile and clarify the role of the GTD to the pathogenesis of CDI, we applied allelic-exchange technology to delete the entire 8133bp *tcdA* gene from the chromosome of *C*. *difficile* 630Δ*erm* [20], as described in materials and methods. Successful deletion was observed by the presence of a circa 2.7kb product following PCR with diagnostic primers flanking *tcdA* (**Fig 1A**). An additional PCR was conducted using internal primers annealing within *tcdA* to ensure the absence of contaminating wild-type sub-populations (**Fig 1B**). The selected mutant, designated 630ΔermΔ*pyrE*Δ*tcdA* possessed the 2.7Kb deletion fragment and lacked the circa 500bp internal fragment following both PCR reactions (**Fig 1A and 1B**). The deletion was confirmed to be as intended through Sanger sequencing of the resultant amplicon. Deletion of *tcdA* should permit the study of TcdB without the need to consider the contribution of TcdA-mediated virulence. Using the same technology, the trinucleotide gac encoding Asp 270 in *tcdB* was mutated to aat to encode Asn ensuing an Asp270Asn (D270N) substitution. TcdB D270N was previously shown to be the most defective variant for glucosyltransferase activity *in vitro*, and was fully capable of inducing the necrotic mechanism of cell death [13]. PCR with flanking primers (**Fig 1C**) followed by Sanger sequencing of the amplicon and sequence alignment using the Benchling bioinformatics platform, revealed that the gac-aat substitution had taken place as intended (**Fig 1D**). Finally, the *pyrE* allele was repaired in both strains exactly as described previously [21]. To probe for unintended polymorphisms arising as a consequence of the mutagenesis procedure, gDNA was extracted from each strain and analyzed by next generation sequencing (NGS). No unintended nucleotide variations were detected for the two mutant strains compared with the wild-type parental. Sequencing reads were deposited to the NCBI Sequencing Read Archive [22] under accession number PRJNA623295.

Before proceeding with the animal experiments, it was first necessary to phenotype the mutants generated herein. The production of TcdA and TcdB was assessed by Western blot on 48h filter-sterilized supernatants of the wild-type and mutant strains. Neither the *tcdA*-deletion strain nor the GT-defective mutant were capable of producing TcdA, as intended (**Fig 1E**). In a similar fashion, neither strain was affected for the production of TcdB (**Fig 1E**). Ponceau staining demonstrated adequate and equal protein loading. The strains were then assessed for their growth capabilities through an automated 24h growth curve. No growth defects were observed for either mutant when compared with the wild-type parental (**Fig 1F**).

### Glucosyltransferase activity is required for disease pathogenesis in a murine model

Having generated the requisite mutants, we next investigated their effects in a non-lethal mouse model of CDI. This model mimics human disease in that gastrointestinal (GI) dysbiosis is first induced with antibiotics, the inciting risk factor in a majority of human infections. Further, a majority of human infections do not result in death, but instead cause pathology in the distal GI tract, similar to the murine model of infection. Therefore, this model serves to help investigate whether GT activity was necessary for clinically relevant measures of TcdB-induced pathogenesis *in vivo*.

**Fig 2:**
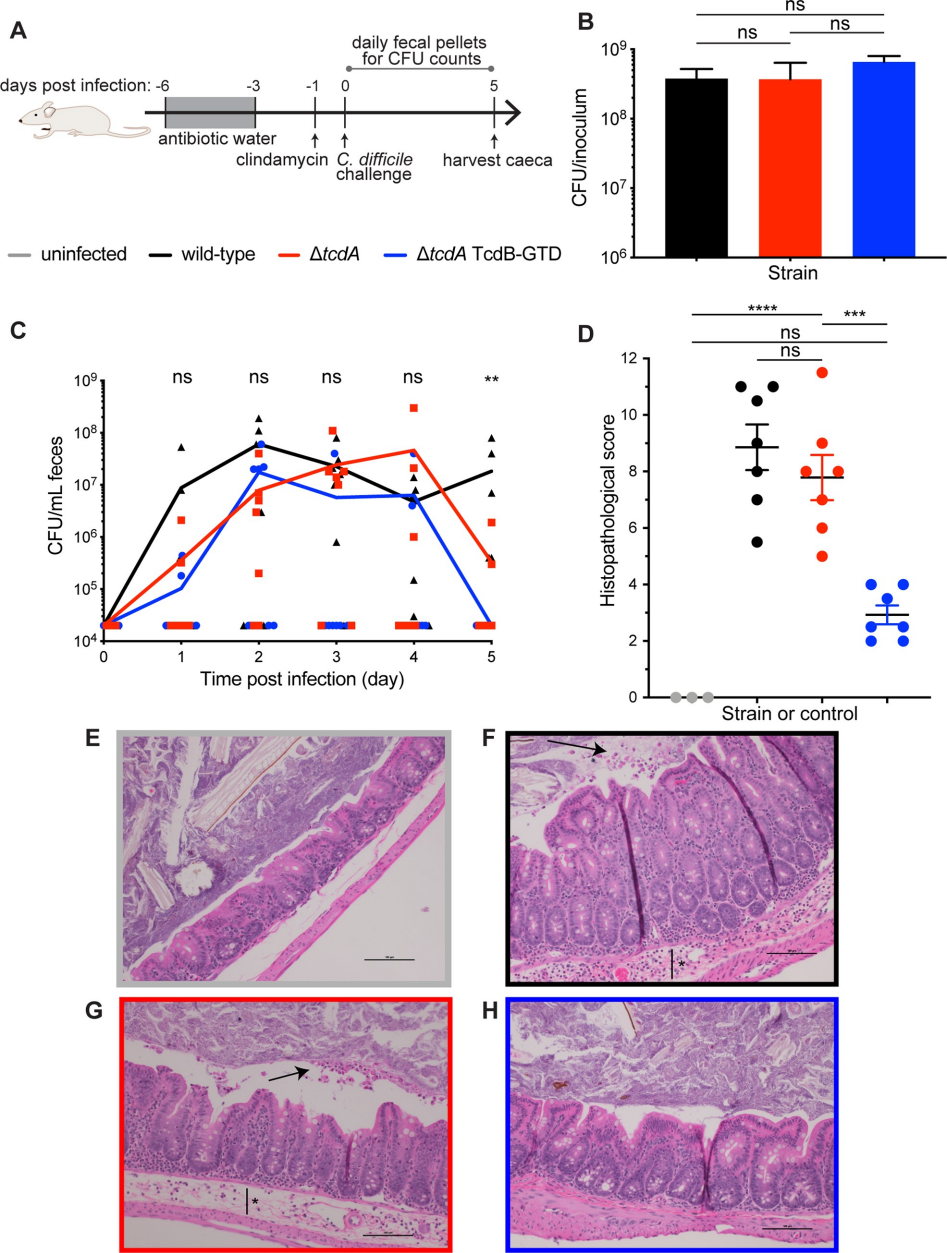
The GTD is required for typhlitis in murine infection. **a)** Schematic of non-lethal mouse model of CDI. Swiss Webster mice, excluding those in the uninfected control group, were pretreated with an antibiotic cocktail in drinking water for three days (gray box, days -6 to -3), then orally dosed with 1 mg clindamycin on day -1 to induce dysbiosis. On day 0, mice were orally challenged with *C*. *difficile* 630Δ*erm* (wild-type, n = 7), *C*. *difficile* 630Δ*erm*Δ*tcdA* (*tcdA* deletion mutant, n = 7), *C*. *difficile* 630Δ*erm*Δ*tcdA* GTD::D270N (GT-defective mutant, n = 7) or sterile PBS for the uninfected control group (n = 3). Fecal pellets were collected daily to analyze *C*. *difficile* burden and caeca were harvested on day 5 for histological analysis. **b)** *C*. *difficile* burden per 200 μL inoculum quantified for each bacterial strain. Statistical analysis between *C*. *difficile* strains was analyzed using one-way ANOVA with Tukey’s multiple comparison test (ns, non-significant). **c)** Daily *C*. *difficile* burden measured from selective culture of fecal samples. Graph displays each replicate with connecting line indicating the mean per group at each time point. Statistical analysis between groups was performed on each day for days 1–4 via Kruskal-Wallis test (ns, non-significant) and on day 5 via Kruskal-Wallis with multiple comparisons (**p<0.01 between wild-type and GT-defective mutant, all other comparisons non-significant). d) Histopathological score for combined inflammatory cell infiltrates (0–3), mucosal hypertrophy (0–3), epithelial disruption (0–3) and submucosal edema (0–3), with each pathological feature scored from normal (0) to severe (3) on H&E-stained caecal slides. Statistical analysis performed via one-way ANOVA with Tukey’s multiple comparison test (ns, non-significant; ***p<0.001, ****p<0.0001). Representative H&E image of caecal section from **e)** uninfected control group, **f)** wild-type group, **g)** *tcdA* deletion mutant group, and **h)** GT-defective mutant group. Images were taken with x20 magnification, and scale bar indicates 100 μM. Black arrows indicate apoptotic cell sloughage, black bar with asterisk indicates submucosal edema.

In this established model, pretreatment with an antibiotic cocktail followed by a single dose of clindamycin was used to disrupt the GI microbiome, creating a GI state permissive to *C*. *difficile* colonization (**Fig 2A**) [23]. Mice were then challenged with approximately 10^8^ colony-forming units (CFUs) of either wild-type *C*. *difficile* 630Δ*erm* (WT), the *tcdA*-deletion mutant *C*. *difficile* 630Δ*erm* (Δ*tcdA*) or the GT-defective strain *C*. *difficile* 630Δ*erm* GTD::D270N (TcdB-GTD). *C*. *difficile* burdens per inoculum, with mean averages of 3.8 × 10^8^ for WT, 3.7 × 10^8^ for Δ*tcdA*, and 6.6 × 10^8^ TcdB-GTD, did not statistically significantly differ between the three strains (**Fig 2B**). To determine whether mutant strains were able to colonize *in vivo*, mice were monitored for 5 days post infection with daily CFU counts from fecal samples. Selective culturing confirmed that each mouse in all three groups shed detectable CFUs on at least one day. Genomic DNA isolated from one fecal sample per cage was amplified to identify the desired strain (**S1A–S1C Fig**). These data confirm that each group was colonized with the administered strain.

Aggregate daily CFUs within each group displayed a wide range, with multiple measurements falling below the limit of detection of 2 × 10^4^ CFU/mL feces (**Fig 2C**). However, statistical analysis between shed CFUs of WT, Δ*tcdA*, and TcdB-GTD on days one to four were not significantly different (**Fig 2C**), indicating similar levels of colonization among the three groups over these days. We noted that on day five, *C*. *difficile* burdens were all below the limit of detection in the TcdB-GTD group, which was significantly less compared to WT (p = 0.0034) and narrowly missing significance as compared to the Δ*tcdA* group (p = 0.0538). While this could suggest that the TcdB-GTD mutant displays different colonization kinetics than the WT and Δ*tcdA* strains, we posit that the variance in these data are likely ascribed to a systematic technical error or the high limit of detection of the method (2 × 10^4^ CFU/mL). Notwithstanding the potential differences in colonization kinetics, these data show that all strains colonized *in vivo*.

Having established that all groups were colonized, we asked whether GT activity was required for evidence of pathogenesis in the distal GI tract. Mice were sacrificed on day 5 post infection to harvest caecal tissue for histological analysis. H&E-stained slides of caecal samples were analysed for evidence of inflammatory cell infiltrates, mucosal hypertrophy, epithelial disruption and submucosal edema, with a maximal severity score of 12 (**Fig 2D**). WT and Δ*tcdA* groups displayed significant evidence of CDI. As compared to uninfected controls (**Fig 2E**), representative images of the WT and Δ*tcdA* groups display apoptotic cell sloughage (**Fig 2F and 2G** black arrows) and submucosal edema (**Fig 2F and 2G** black bar with asterisk). Histopathological scores were not significantly different between the WT and Δ*tcdA* groups (p = 0.6537). Thus, the absence of TcdA does not significantly alter disease severity, consistent with prior studies [4, 5]. In contrast, the TcdB-GTD group displayed significantly attenuated pathology, with scores significantly lower than WT (p<0.0001) and Δ*tcdA* (p = 0.0002), and lacking evidence of severe disease in representative images (**Fig 2H**). Further, pathology in the TcdB-GTD group was not significantly different than uninfected controls (p = 0.0957). Taken together, these data confirm that GT activity is required for pathogenesis in a clinically relevant model of CDI.

### Glucosyltransferase activity is required for *C*. *difficile* pathogenesis in a hamster model

Having established that GT activity is required for pathogenesis in a non-lethal model of CDI, we next asked whether the TcdB-GTD mutant strain was capable of inducing virulence in a more sensitive animal model. To test this, we used the well-established hamster model of infection. Unlike the non-lethal mouse model of CDI, hamsters are exquisitely sensitive to *C*. *difficile* and uniformly succumb to fulminant infection when challenged with *C*. *difficile* expressing wild-type toxin, but survive when challenged with avirulent strains [4]. As a consequence, we reasoned that the hamster model could potentially reveal any symptoms of CDI which were not detected in the less sensitive murine model.

**Fig 3:**
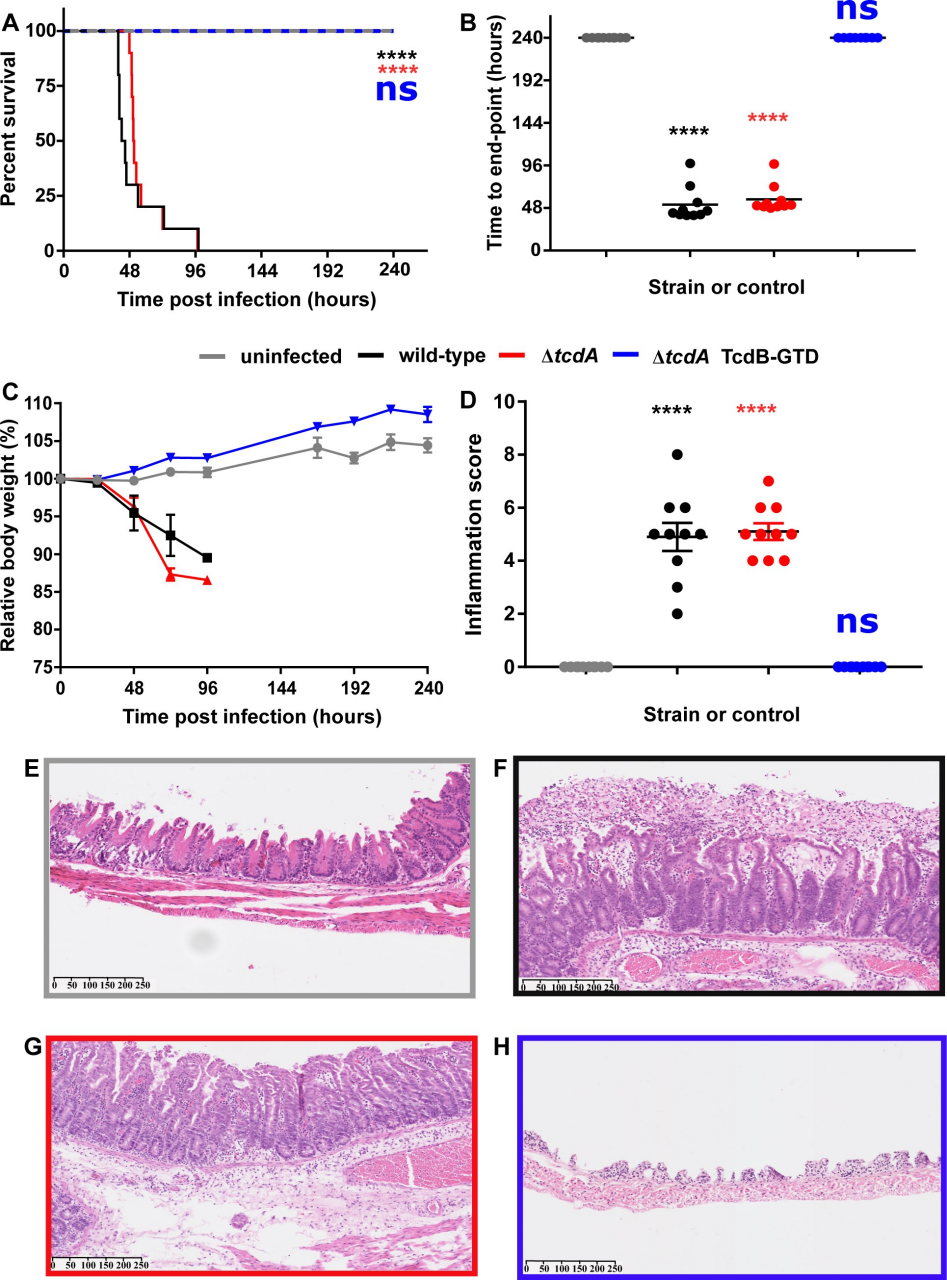
The GTD of TcdB is indispensable for *in vivo* pathogenesis in hamsters. **a)** Survival plot of Syrian Golden hamsters infected with approximately 10,000 spores of *C*. *difficile* 630Δ*erm* (wild-type, black lines), *C*. *difficile* 630Δ*erm*Δ*tcdA* (*tcdA* deletion mutant, red lines), *C*. *difficile* 630Δ*erm*Δ*tcdA* GTD::D270N (GT-defective mutant, blue lines) or sterile PBS for the uninfected control group (grey lines) (n = 10). Hamsters were monitored for a period of 240h for primary symptoms of CDI. Animals were euthanized at the onset of fulminant infection, or at the 240h time-point in the absence of considerable symptoms. Statistical significance between test groups and the uninfected control group was shown according to the Peto log-rank test (p = **** <0.0001) **b)** Time from inoculation to experimental endpoint. Statistical significance between test groups and the uninfected control group according to one-way ANOVA followed by Dunnet’s multiple comparison test (p = **** 0.001) **c)** Body weight of each animal subject, relative to starting weight. Data points represent the mean ±SEM of 10 animal subjects per infection or control group **d)** Terminal inflammation score for combined edema (0–3), tissue damage (0–3) and neutrophil infiltration (0–3) (maximum score of 9) as assessed by histopathological analysis of H&E stained caeca. Statistical significance between test group and the uninfected control group according to one-way ANOVA followed by Dunnet’s multiple comparison test (p = **** 0.0001). Representative transverse cross-section of H&E stained hamster caeca for **e)** Uninfected control group **f)** *C*. *difficile* 630Δ*erm* (wild-type group) **g)** *C*. *difficile* 630Δ*erm*Δ*tcdA* (tcdA deletion group) **h)** *C*. *difficile* 630Δ*erm*Δ*tcdA* GTD::D270N (GT-defective group). Scale bars represent the μM range.

To this end, pre-conditioned Syrian Golden hamsters were infected with approximately 10,000 heat-resistant colony-forming units (HR-CFU) of either WT, Δ*tcdA* or TcdB-GTD. The uninfected control group was given sterile diluent at time-point 0. Ten animals were used per infection group or control. Hamsters were monitored at regular intervals for primary symptoms of CDI (severe wet tail, diarrhea, hypothermia, lying prone or unresponsive) and were euthanized at the onset of fulminant CDI or at the 240^th^ hour post infection. Five animals infected with wild-type 630Δ*erm* were symptomatic of fulminant CDI 36h following spore administration (**Fig 3A**). The remaining hamsters in this group reached this stage by the 96h time-point. The mean time to end-point following spore administration was 52h for the WT group (**Fig 3B**). Similarly, animals infected with the Δ*tcdA* strain reached fulminant CDI with an average time to end-point of 58h (**Fig 3B**). All animals in WT and Δ*tcdA* groups succumbed to fulminant infection by 96h (**Fig 3A and 3B**). Deletion of *tcdA* did not significantly reduce the time from spore administration to end point (P = 0.94), thus further supporting the centrality of TcdB to disease pathogenesis. Hamsters in both the WT and Δ*tcdA* groups lost significant weight, up to 12.5% relative to their starting weights (**Fig 3C**). Meanwhile, none of the subjects infected with the TcdB-GTD strain presented with any primary symptom of CDI during the 240h infection period. Consequently, all subjects in this group were euthanized at the 240h time-point (**Fig 3A and 3B**). Contrary to the results seen for the WT and Δ*tcdA* animals, subjects within this group gained up to 8% body weight relative to their starting weight (**Fig 3C**). Terminal colonization was assessed as described in Materials and Methods. Each infection group was colonized by *C*. *difficile* (**S2 Fig**). The approximately 2-log decrease observed between the 24-96h samples for the WT and Δ*tcdA* groups compared with the 240h GT-defective group, are in-line with previous 10-day colonization experiments [24]. Owing to the sampling procurement adopted, it is not possible for us to demonstrate that each mutant colonized the hamsters to the same extent. However, the NGS data assures us that the GT-defective mutant was devoid of unintended mutations which may have affected colonization. Taken together, these data indicate that the GTD is essential for the development of primary symptoms during CDI.

Having established that the GTD is required for the onset of primary symptoms for CDI, we next tested for any signs of inflammation induced by each respective mutant through histopathological analysis of hamster caeca. Both the WT and Δ*tcdA* groups showed significant signs of caecal inflammation yielding similar inflammation scores of approximately 5 based on the sum of edema, neutrophil infiltration and tissue damage, with a maximum score of 9 per animal subject (**Fig 3D, 3F and 3G**). On the contrary, the subjects in the uninfected control group and the TcdB-GTD groups showed no symptoms of inflammation yielding a score of zero for each individual subject (**Fig 2D, 2E and 2H**). The results from the histopathological analysis corroborate those of the primary infection study thus suggesting that the GTD is required for virulence in TcdB-induced CDI.

### Concluding remarks

The data presented herein, in two well-established animal models of CDI, demonstrate that GT activity is essential for *in vivo C*. *difficile* disease pathogenesis. Although recombinant GT-defective TcdB is clearly capable of inducing cellular toxicity *in vitro* at higher nanomolar concentrations through both necrotic or pyknotic mechanisms [13, 15], the lack of any overt disease pathology for the GT-defective TcdB strain in either model tested here, suggests that the absolute amounts of toxin needed to induce these cellular phenotypes may not be reached during an infection *in vivo*. Future studies on the role of GT activity in other models and other aspects of disease such as recurrence and colonization kinetics are warranted to determine whether GT-independent effects play a role in other aspects of CDI.

Finally, the results presented here have important implications on our understanding of CDI disease pathogenesis and obvious consequences for the design of novel SMIs aiming to neutralize the toxic effects of TcdB as an alternative to antibiotic therapy. Before the discovery of the necrotic and pyknotic mechanisms of toxicity, the major focus for SMI development was focused on identification of GTD-targeted SMIs. Our demonstration that the GTD is essential for the development of symptoms during CDI, reoffers the GTD as a well-validated target for SMI development.

## Materials & methods

### Ethics statement

All murine experiments were carried out in accordance with APLAC protocols approved by the Stanford University Institutional Care and Use Committee (IACUC).

### Bacterial growth conditions

For strain development and *in vitro* analyses, *C*. *difficile* was routinely maintained on Brain heart infusion medium (Oxoid) supplemented with 5μg/ml yeast extract, 0.1% w/v L-cysteine (BHIS), *C*. *difficile* selective supplement comprising 250μg/ml D-cycloserine, and 8 μg/ml cefoxitin (Oxoid) (BHIS CC), and where necessary, an additional supplementation of 15 μg/ml thiamphenicol (BHIS CCTM). *C*. *difficile* cultures were grown at 37°C in an anaerobic workstation (Don Whitley) with an anaerobic gas mixture comprising 80% N_2_, 10% H_2_ and 10% CO_2_.

For murine experiments, frozen stocks of wild-type and mutant strains were cultured on CDMN agar plates (*C*. *difficile* agar base (Oxoid) supplemented with 7% (v/v) defibrinated horse blood (Lampire Biological Laboratories), 32 mg/L moxalactam (Santa Cruz Biotechnology), 12 mg/L norfloxacin (Sigma-Aldrich) and 500 mg/L cysteine hydrochloride (Fischer) in an anaerobic chamber [27] at 37°C for 24 hours. Single colonies were picked and grown anaerobically for 16–18 hours at 37°C to saturation in 5 mL of pre-reduced reinforced Clostridial medium (RCM, Oxoid) for inoculation.

For hamster experiments, strains were cultured on blood agar for 5d in order to generate the spore stocks. Terminal colonization was assessed using *C*. *difficile* selective agar (Oxoid). In both instances, strains were maintained in an anaerobic workstation, as described above.

### Mutant generation

Strains and primers used in this study are listed in Tables 1 and S1 respectively. An in-frame deletion mutant of *tcdA* in 630Δ*erm*Δ*pyrE* was generated using allelic-exchange technology [20]. Left and right homology arms annealing up and downstream of *tcdA* were amplified by PCR with primer sets *tcdA* LAF/LAR and RAF/RAR respectively. The fragments were spliced together by splicing by overlap extension (SOEing) PCR and were subsequently cloned into pMTL-YN3 [20], by means of their flanking *Sbf*I and *AscI* restriction sites. The Knockout cassette [28] was conjugated into 630ΔermΔ*pyrE* exactly as described previously [21]. Transconjugants were selected on the basis of thiamphenicol resistance on BHIS CCTM plates. Single cross-over integrants were identified following sub-culturing onto BHIS CCTM by PCR using two primer sets to screen for integration at the left or right homology arms: *tcdA* diag F/YN3 R and YN3 F/ *tcdA* diag R; each containing a primer annealing to the chromosome and a primer annealing to the KOC plasmid. Confirmed single cross-over clones were grown on non-selective BHIS medium and thereafter *C*. *difficile* minimal medium [29] containing 500μg/ml 5-fluoroorotic acid (FOA) and 1μg/ml uracil to force plasmid loss through the counter-selection marker *pyrE*, and to select for double cross-over mutants before confirming plasmid loss on the basis of thiamphenicol sensitivity. The desired deletion mutants were identified by PCR amplification of the *tcdA* locus using the diagnostic primers *tcdA* diag F and *tcdA* diag R. An additional PCR was conducted on putative mutants with internal primers annealing within *tcdA* to ensure the absence of contaminating wild-type sub-populations.

**Table 1:** Strains used in this study.

Strain/Plasmid	Description	Origin
Strain		
*E*. *coli*		
Top 10	Cloning host	Invitrogen
CA434	Conjugation host	[25]
*C*. *difficile*		
630Δ*erm*	Erythromycin sensitive clone of CD630	[26]
630Δ*erm*Δ*pyrE*	*pyrE* mutant to facilitate AE mutagenesis	[20]
630Δ*erm*Δ*pyrE*Δ*tcdA*	Initial *tcdA*-deletion mutant	This study
630Δ*erm*Δ*tcdA*	*pyrE*-restored *tcdA*-deletion mutant	This study
630Δ*erm*Δ*pyrE*Δ*tcdA*.TcdB::GTD D270N	Initial GTD-defective mutant	This study
630Δ*erm*Δ*tcdA*.TcdB::GTD D270N	*pyrE*-restored GTD-defective mutant	This study
Plasmid		
pMTL-YN3	Knockout vector for 630Δ*erm*	[20]
pMTL-YN3-tcdA KOC	As above with knockout cassette for *tcdA*	This study
pMTL-YN3-TcdB::GTD D270N SC	As above with substitution cassette for TcdB::GTD D270N	This study
pMTL-YN1	*pyrE*-restoration vector for 630Δ*erm*	[20]

For the generation of a GTD-defective mutant, two circa 1kb fragments were amplified by two PCR reactions for each target, comprising a forward and reverse primer containing *SbfI* and *AscI* restriction sites respectively, paired with internal mutagenic primers encompassing the desired nucleotide substitutions. The mutagenic forward primer GTD mut F, contained the exact reverse complemented sequence of the mutagenic reverse primer, GTD mut R to facilitate subsequent SOEing of the two fragments. Spliced products were then cloned into pMTL-YN3 as described above to generate a substitution cassette (SC) for GTD D270N. The procedure hereafter mimicked that described above for the chromosomal deletion, however, owing to the desired mutagenesis being a substitution rather than a deletion, putative mutants were assessed by PCR followed by Sanger sequencing of the amplicon. The read for each clone was aligned to the wild-type reference sequence and the desired substitution sequence to confirm the presence or absence of the desired codon substitution. The *pyrE* locus of each strain was then restored exactly as described previously [21].

### Whole genome sequencing

Genomic DNA was extracted from both mutant strains and the wild-type parental and sent for next generation sequencing (NGS) using the Illumina MiSeq platform at MicrobesNG (University of Birmingham, UK). The paired reads were trimmed and mapped to the reference genome sequence for *C*. *difficile* 630 (Accession: AM180355. Thereafter, each strain was analyzed for any single nucleotide variations (SNVs), insertions or deletions of DNA compared with the reference genome sequence, using CLC Genomics Workbench software (Qiagen, Netherlands). Sequencing reads were deposited to the NCBI Sequencing Read Archive under accession number PRJNA623295.

### Western blot detection of *C*. *difficile* toxins

Secreted TcdA and TcdB was detected by Western blot on 40x trichloracetic acid protein concentrates derived from 48h sterile-filtered supernatants exactly as described previously [21] using HRP-Chicken anti-*Clostridium difficile* Toxin A IgY and anti-*Clostridium difficile* Toxin B IgY antibodies (Gallus-Immunotech, USA) respectively.

### Automated growth curve

Growth profiles of the wild-type parental and mutant strains were assessed by automated growth curve using a Promega Glomax plate reader (Promega, USA) exactly as described previously [30].

### Murine model of infection

Age- and sex-matched male and female conventional Swiss Webster mice (SWEF, Taconic, bred in-house) ages 8–12 weeks were fed standard chow and had an average starting weight of 38 g. Mice were randomly divided into four groups: uninfected controls without antibiotic pre-treatment (n = 3), or infected with WT (n = 7), *ΔtcdA* (n = 7) or TcdB-GTD (n = 7). Mice, excluding those in the control group, were pre-treated with an antibiotic cocktail (kanamycin (0.4 mg/mL), gentamycin (0.035 mg/mL), colistin (850 U/mL), metronidazole (0.215 mg/mL) and vancomycin (0.045 mg/mL)) in drinking water for 3 days, starting 6 days before inoculation, as previously reported [23]. Mice were switched to regular water for 2 days, and then orally administered 1 mg of clindamycin on day -1 (excluding those in the uninfected control group). On day 0, experimental WT, *ΔtcdA* and TcdB-GTD were orally challenged with approximately 10^8^ CFU (in 200 μL RCM) of the appropriate wild-type or mutant strain from overnight culture. Mice were monitored with daily weights, and fecal samples were collected for 5 days to measure daily *C*. *difficile* CFU burden. Mice were then sacrificed according to the guidelines on human termination on experimental day 5 after *C*. *difficile* challenge. Caecal tissues were collected for histological analysis. Genomic DNA from fecal samples from one mouse per cage was amplified to ensure colonization with the appropriate strain, using a protocol adapted from [31, 32]. Briefly, 10–30 mg fecal sample was resuspended in 500–700 μL TRIS-buffered saline (TBS, 50 mM TRIS, 150 mM NaCl, pH 7.4) and disrupted with 0.1 mm glass beads using Mini-BeadBeater-96 (Biospec Products) for 3 minutes. Genomic DNA was extracted from the supernatant with two rounds of phenol:chloroform:isoamyl alcohol (25:24:1) in a 1:1 ratio with supernatant, then ethanol precipitation was performed. The resulting DNA pellet was resuspended in 200 μL PBS and purified of PCR inhibitors using a QIAamp DNA blood Mini kit (Qiagen) before amplifying the regions of interest using *tcdA* int F and R primers (TcdA internal region) and GTD diag F and R primers (GTD-encoding region of TcdB).

### Fecal and Inoculum and CFU quantification in the murine model

Fecal samples were collected from mice directly into Eppendorf tubes and placed on ice. To measure *C*. *difficle* CFU burdens, 1 μL feces was resuspended in 200 μL sterile PBS. Samples were then serially diluted 10-fold into PBS, with 10 μL struck onto CDMN plates in duplicate. Plates were incubated anaerobically at for 16–24 hours at 37°C before quantification. To enumerate inoculum CFUs, 200 μL of each strain was used as starting material, with serial dilution and plating as above.

### Histopathological assessment of murine samples

Proximal colon and caecal sections were collected and fixed in 10% neutral buffered formalin, processed (Leica), paraffin embedded (Leica), sectioned at 5 μm, mounted on glass slides and H&E stained (HistoTec Laboratory). Sections were scored by a veterinary pathologist blinded to the identity of the samples (Dr. Donna M. Bouley, Stanford University Department of Comparative Medicine) using a scoring system adapted from Pawlowski et al [33]. Caecal sections were assessed for inflammatory cell infiltrates (0–3), mucosal hypertrophy (0–3), epithelial disruption (0–3) and submucosal edema (0–3) for a maximal score of 12, with each pathological feature scored from normal (0) to severe (3). Images representative of each treatment group are shown in Fig 2.

### Hamster model of infection

Animal studies were planned and performed according to the ARRIVE guidelines [34] under license by Evotec Ltd, UK. Adult male Syrian Golden hamsters weighing between 82-111g were pre-conditioned with 30mg/kg clindamycin to disrupt their gut microbiota for a period of 5d. Hamsters were then randomly divided into four groups: uninfected controls (n = 10), or infected with WT (n = 10), *ΔtcdA* (n = 10) or TcdB-GTD (n = 10). Subjects in each experimental group were administered with approximately 10,000 spores (heat-resistant colony forming units, HR-CFU) of the appropriate wild-type or mutant strain or sterile PBS (uninfected control group). Animals were monitored daily for symptoms of CDI for a period of 240h. Animals were euthanized by pentobarbitone overdose, if they were deemed to be symptomatic of fulminant CDI. Criteria for fulminant CDI were: hypothermia <34°C, 20% weight loss, severe diarrhea, inability to reach food and water or severely hunched posture with piloerection. Euthanized subjects were dissected and terminal-colonization was determined in-house at the animal research facility using fecal samples plated on *C*. *difficile* selective medium (Oxoid, USA). The presence of *C*. *difficile* was confirmed using the *C*. *difficile* latex agglutination kit (Oxoid, USA). To confirm the identity of the isolates, HR-CFU were isolated from caecum, small intestine and colon samples in the research facility. Presumptive isolates were confirmed by colony PCR for *tcdA* flanking region, *tcdA* internal region and GTD-encoding region of *tcdB*. Hamster caeca were fixed in 10% buffered neutral formalin (BNF) and stored until histopathological assessment.

### Histopathological assessment of hamster caeca

Fixed transverse cross-sections of hamster caeca were routinely processed for paraffin embedding, sectioned at 5 μm, mounted on glass slides, and H&E stained. Thereafter a trained pathologist individually scored each sample for edema (1–3), neutrophil infiltration (1–3) and tissue damage (1–3), with a maximum potential score of 9 per animal subject.

### Statistical analysis

Statistical analyses (one-way ANOVA with Tukey’s or Dunnet’s multiple comparison test, Kruskall-Wallis test with multiple comparisons, as indicated in figure legends) were performed using the GraphPad Prism 7 and 8 software (GraphPad Software).

## Supporting information

S1 Table Oligonucleotide primers used in this study. (PDF)

S1 Fig Genomic isolation and PCR confirms appropriate strains from feacal samples in mouse model of CDI. **a)** Gel image following genomic isolation of DNA from fecal samples and PCR of the *tcdA* gene using the internal primers *tcdA* int F/*tcdA* int R. The absence of a 500bp product indicates Δ*tcdA* isolate. **b)** Gel image following genomic isolation of DNA from fecal samples and PCR of the GTD of *tcdB* using GTD diag F/R primers. Strain labels: *C*. *difficile* 630Δ*erm* (wild-type) cages 1 and 2; *C*. *difficile* 630Δ*erm*Δ*tcdA* (Δ*tcdA*) cages 1 and 2; *C*. *difficile* 630Δ*erm*Δ*tcdA* GTD::D270N (Δ*tcdA* TcdB-GTD) cages 1 and 2. **c)** Sequence alignment of the GTD region of *tcdB* for *C*. *difficile* 630Δ*erm*Δ*tcdA* GTD::D270N with the wild-type reference sequence of 630Δ*erm*. The gac-aat substitution is highlighted in a red box.
(PDF)

S2 Fig Hamster subjects are sufficiently colonized by *C*. *difficile*. Terminal burden (heat-resistant colony-forming units/g HR-CFU/g) of *C*. *difficile* for uninfected, WT, *ΔtcdA* and TcdB-GTD infection groups. Spores were isolated from fecal samples at the day of experimental end-point for each animal subject. Since the burden was determined at different time-points for each animal subject, it is not appropriate to perform statistical analyses for these data.
(PDF)
