# Supplementary material for: The glucosyltransferase activity of C. difficile Toxin B is required for disease pathogenesis
Source: PLoS Pathog. 2020 Sep 22;16(9):e1008852. doi: 10.1371/journal.ppat.1008852 (PMC7531778; doi:10.1371/journal.ppat.1008852)
Supplement: S1 Table — (PDF) [file ppat.1008852.s001.pdf]

Table S1: Oligonucleotide primers

**Primer**	**Sequence 5'-3'**
***tcdA* deletion**	
tcdA KOC LAF	TTTTTTCCTGCAGGGTTTTGGAATAGATGGAGGAATCAG
tcdA KOC LAR	ACATATATTTTAGCCAGACATAAAAACCTCCTAGTATTATTATTTTTG
tcdA KOC RAF	GAGGTTTTTATGTCTGGCTAAAATATATGTTTGATAAAAAATTATTC
tcdA KOC RAR	AAAAAAGGCGCGCCGATGGTAACGAATTTAGTAATGAAGG
tcdA diag F	GGTGGACTATGATGAATGCAC
tcdA diag R	CACCTCTAAATCACTGAGTC
tcdA int F	GGAGAAGTCAGTGATATTGC
tcdA int R	GATGCTGCAGCTAAATTTCC
**GTD D270N substitution**	
GTD F	TTTTTTCCTGCAGGAACGTCTTTATTCAATCGAAGAGC
GTD mut R	CAGATATTCTTAATAT**ATT**AGAAGCAGCAG
GTD mut F	CTGCTGCTTCT**AAT**ATATTAAGAATATCTG
GTD R	AAAAAAGGCGCGCCTTGCAAATAAGTTACATGCTGC
GTD SCO F	CTTGTAATTAATGAGCTTAAAG
GTD diag F	GGAGATAGATGAACTTAATACC
GTD diag R	GCTTCTAACTTTGTCATTTCC
**SCO determination**	
**YN3 F**	CTCCATCAAGAAGAGCGAC
**YN3 R**	CTTTCTATTCAGCACTGTTATGCC
***pyrE* restoration**	
pyrE F	GGAGCTACTTGTATCCAAG
pyrE R	CCTAATTCCTTGAACTCTC
