# Supplementary figures and images for: The glucosyltransferase activity of C. difficile Toxin B is required for disease pathogenesis
Source: PLoS Pathog. 2020 Sep 22;16(9):e1008852. doi: 10.1371/journal.ppat.1008852 (PMC7531778; doi:10.1371/journal.ppat.1008852)

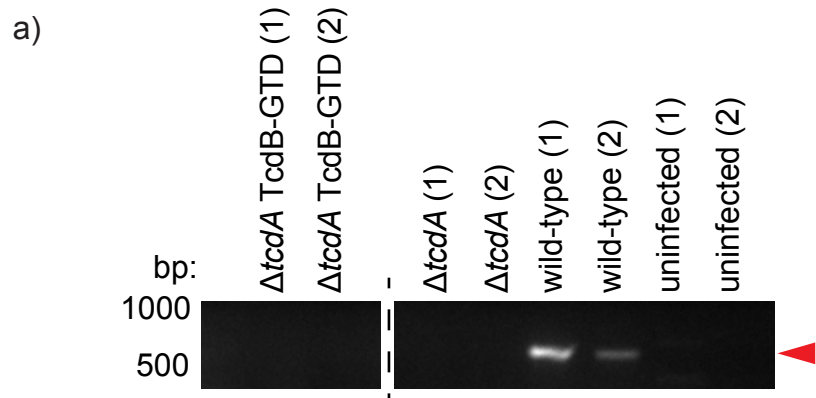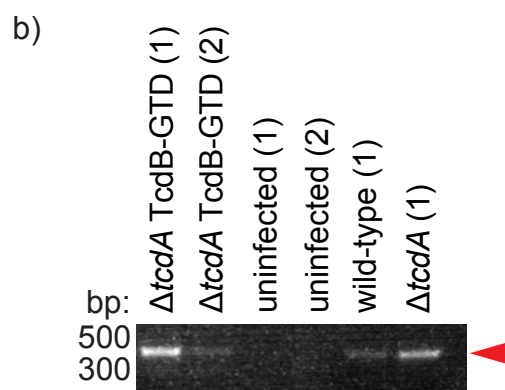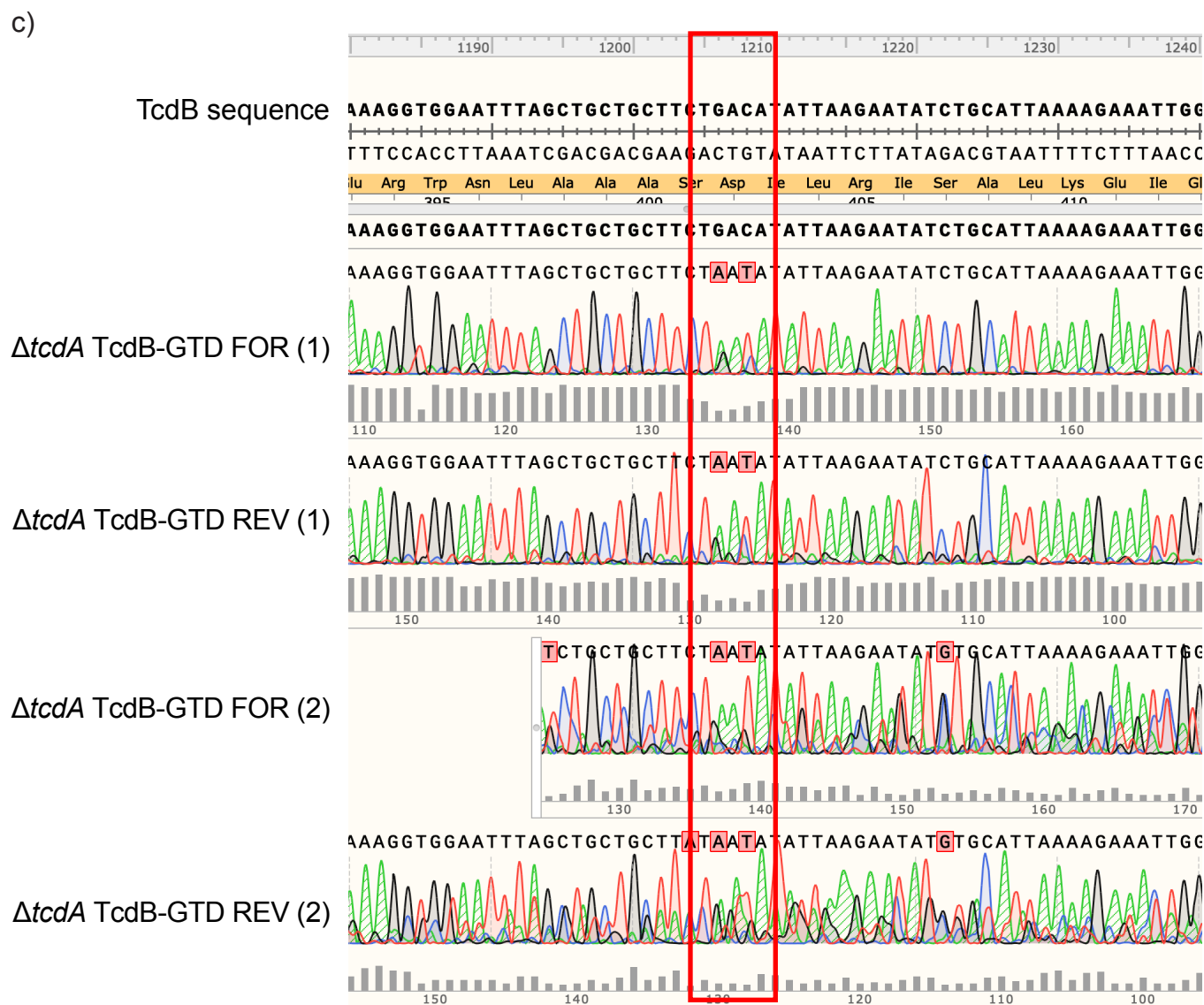

Supplement: S1 Fig — a) Gel image following genomic isolation of DNA from fecal samples and PCR of the tcdA gene using the internal primers tcdA int F/tcdA int R. The absence of a 500bp product indicates ΔtcdA isolate. b) Gel image following genomic isolation of DNA from fecal samples and PCR of the GTD of tcdB using GTD diag F/R primers. Strain labels: C. difficile 630Δerm (wild-type) cages 1 and 2; C. difficile 630ΔermΔtcdA (ΔtcdA) cages 1 and 2; C. difficile 630ΔermΔtcdA GTD::D270N (ΔtcdA TcdB-GTD) cages 1 and 2. c) Sequence alignment of the GTD region of tcdB for C. difficile 630ΔermΔtcdA GTD::D270N with the wild-type reference sequence of 630Δerm. The gac-aat substitution is highlighted in a red box. (PDF) [file ppat.1008852.s002.pdf]

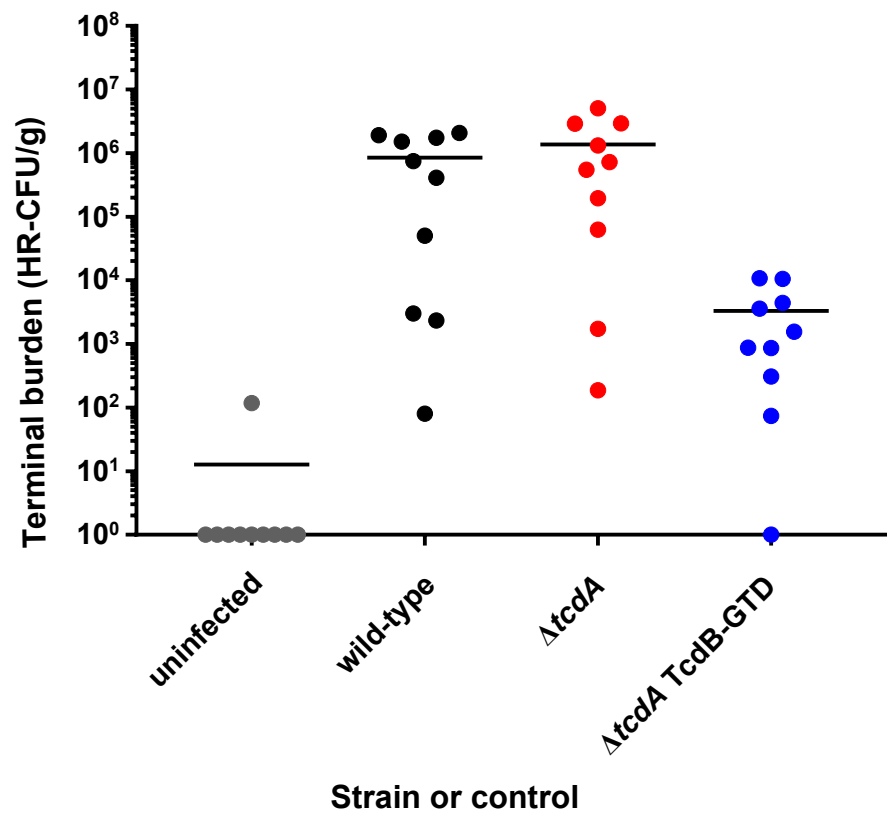

Supplement: S2 Fig — Terminal burden (heat-resistant colony-forming units/g HR-CFU/g) of C. difficile for uninfected, WT, ΔtcdA and TcdB-GTD infection groups. Spores were isolated from fecal samples at the day of experimental end-point for each animal subject. Since the burden was determined at different time-points for each animal subject, it is not appropriate to perform statistical analyses for these data. (PDF) [file ppat.1008852.s003.pdf]
